# Supplementary material for: Validity of the French version of the Autonomy Preference Index and its adaptation for patients with advanced cancer
Source: PLoS One. 2020 Jan 14;15(1):e0227802. doi: 10.1371/journal.pone.0227802 (PMC6959662; doi:10.1371/journal.pone.0227802)
Supplement: S1 Questionnaire — (DOCX) [file pone.0227802.s001.docx]

**Supporting information S1**

**S1-Questionnaire:** French version of the Autonomy Preference Index (API)

**Questionnaire sur les souhaits d’information et de participation aux décisions concernant la santé**

**(API : Autonomy Preference Index^[[1]](#footnote-1)^)**

**Partie 1 : Participation aux décisions**

1. Les décisions médicales importantes doivent être prises par votre médecin, et non par vous.

Pas du tout d’accord 🞎

Pas d’accord 🞎

Ni d’accord, ni pas d’accord 🞎

D’accord 🞎

Tout à fait d’accord 🞎

1. Il vaut mieux suivre les conseils de votre médecin même si vous n’êtes pas d’accord.

Pas du tout d’accord 🞎

Pas d’accord 🞎

Ni d’accord, ni pas d’accord 🞎

D’accord 🞎

Tout à fait d’accord 🞎

1. Lorsque vous êtes hospitalisé(e), vous souhaitez ne pas avoir à prendre de décision concernant vos soins.

Pas du tout d’accord 🞎

Pas d’accord 🞎

Ni d’accord, ni pas d’accord 🞎

D’accord 🞎

Tout à fait d’accord 🞎

1. Vous souhaitez vous sentir libre de prendre des décisions concernant vos soucis de santé courants.

Pas du tout d’accord 🞎

Pas d’accord 🞎

Ni d’accord, ni pas d’accord 🞎

D’accord 🞎

Tout à fait d’accord 🞎

1. Lorsque vous êtes malade et que votre maladie s’aggrave, vous souhaitez que votre médecin décide davantage pour vous des soins appropriés.

Pas du tout d’accord 🞎

Pas d’accord 🞎

Ni d’accord, ni pas d’accord 🞎

D’accord 🞎

Tout à fait d’accord 🞎

1. Vous souhaitez décider vous-même de la fréquence de vos bilans de santé.

Pas du tout d’accord 🞎

Pas d’accord 🞎

Ni d’accord, ni pas d’accord 🞎

D’accord 🞎

Tout à fait d’accord 🞎

**Partie 2 : Situations cliniques**

***Les cas présentés dans les encadrés suivants n’ont aucun lien avec votre état de santé. En vous imaginant dans chaque situation décrite, pouvez-vous répondre aux questions suivantes ?***

Supposez que vous ayez mal à la gorge, le nez bouché, et de la toux depuis trois jours. Vous êtes sur le point d’appeler votre médecin traitant.

1. Qui doit décider si vous devez être vu(e) par le médecin ?

Seulement vous 🞎

Principalement vous 🞎

Autant le médecin que vous 🞎

Principalement le médecin 🞎

Seulement le médecin 🞎

1. Qui doit décider si vous devez passer une radio des poumons ?

Seulement vous 🞎

Principalement vous 🞎

Autant le médecin que vous 🞎

Principalement le médecin 🞎

Seulement le médecin 🞎

1. Qui doit décider si vous devez essayer de prendre un sirop contre la toux ?

Seulement vous 🞎

Principalement vous 🞎

Autant le médecin que vous 🞎

Principalement le médecin 🞎

Seulement le médecin 🞎

Supposez que vous alliez chez votre médecin pour une visite de routine et que tout aille bien en dehors de votre tension qui est anormalement élevée.

1. Qui doit décider de la date de votre prochaine consultation pour contrôler votre tension ?

Seulement vous 🞎

Principalement vous 🞎

Autant le médecin que vous 🞎

Principalement le médecin 🞎

Seulement le médecin 🞎

1. Qui doit décider de la nécessité d’un arrêt de travail pour vous reposer ?

Seulement vous 🞎

Principalement vous 🞎

Autant le médecin que vous 🞎

Principalement le médecin 🞎

Seulement le médecin 🞎

1. Qui doit décider du choix de votre traitement : prendre un médicament ou suivre un régime ?

Seulement vous 🞎

Principalement vous 🞎

Autant le médecin que vous 🞎

Principalement le médecin 🞎

Seulement le médecin 🞎

Supposez que vous ayez depuis près d’une heure une douleur intense dans la poitrine suffisamment inquiétante pour que vous vous rendiez aux urgences. Aux urgences, les médecins constatent que vous faîtes une crise cardiaque et vous êtes transféré(e) en unité de soins intensifs.

1. Qui doit décider de la fréquence à laquelle les infirmières doivent vous réveiller pour prendre votre température et votre tension ?

Seulement vous 🞎

Principalement vous 🞎

Autant le médecin que vous 🞎

Principalement le médecin 🞎

Seulement le médecin 🞎

1. Qui doit décider de la possibilité de recevoir des visites en plus de celles de votre famille proche ?

Seulement vous 🞎

Principalement vous 🞎

Autant le médecin que vous 🞎

Principalement le médecin 🞎

Seulement le médecin 🞎

1. Qui doit décider de demander l’avis d’un second cardiologue ?

Seulement vous 🞎

Principalement vous 🞎

Autant le médecin que vous 🞎

Principalement le médecin 🞎

Seulement le médecin 🞎

**Partie 3 : Souhaits d’information**

1. Lorsque vous êtes malade et que votre maladie s’aggrave, vous souhaitez être de plus en plus informé(e) sur votre maladie.

Pas du tout d’accord 🞎

Pas d’accord 🞎

Ni d’accord ni pas d’accord 🞎

D’accord 🞎

Tout à fait d’accord 🞎

1. Vous souhaitez comprendre précisément ce qui se passe dans votre corps du fait de la maladie.

Pas du tout d’accord 🞎

Pas d’accord 🞎

Ni d’accord ni pas d’accord 🞎

D’accord 🞎

Tout à fait d’accord 🞎

1. Vous souhaitez être informé(e) même si les nouvelles sont mauvaises.

Pas du tout d’accord 🞎

Pas d’accord 🞎

Ni d’accord ni pas d’accord 🞎

D’accord 🞎

Tout à fait d’accord 🞎

1. Votre médecin doit vous expliquer les objectifs des examens sanguins qu’il vous prescrit.

Pas du tout d’accord 🞎

Pas d’accord 🞎

Ni d’accord ni pas d’accord 🞎

D’accord 🞎

Tout à fait d’accord 🞎

1. Vous préférez recevoir des informations seulement si vous le demandez.

Pas du tout d’accord 🞎

Pas d’accord 🞎

Ni d’accord ni pas d’accord 🞎

D’accord 🞎

Tout à fait d’accord 🞎

1. Il est important pour vous de connaître tous les effets secondaires de votre traitement.

Pas du tout d’accord 🞎

Pas d’accord 🞎

Ni d’accord ni pas d’accord 🞎

D’accord 🞎

Tout à fait d’accord 🞎

1. Vous attachez autant d’importance à l'information sur la maladie qu’à son traitement.

Pas du tout d’accord 🞎

Pas d’accord 🞎

Ni d’accord ni pas d’accord 🞎

D’accord 🞎

Tout à fait d’accord 🞎

1. Lorsqu’il existe plusieurs options de traitement, vous souhaitez être informé(e) sur chacun d’eux.

Pas du tout d’accord 🞎

Pas d’accord 🞎

Ni d’accord ni pas d’accord 🞎

D’accord 🞎

Tout à fait d’accord 🞎

1. Ende J, Kazis L, Ash A, Moskowitz MA. Measuring patients' desire for autonomy: decision making and information-seeking preferences among medical patients. J Gen Intern Med. 1989;4(1):23-30. [↑](#footnote-ref-1)
