# Supplementary material for: Validity of the French version of the Autonomy Preference Index and its adaptation for patients with advanced cancer
Source: PLoS One. 2020 Jan 14;15(1):e0227802. doi: 10.1371/journal.pone.0227802 (PMC6959662; doi:10.1371/journal.pone.0227802)
Supplement: S2 Questionnaire — (DOCX) [file pone.0227802.s002.docx]

**Supporting information S2**

**S2-Questionnaire:** Additional clinical vignette developed for use among patients with incurable cancer, to assess their preparedness to anticipate disease worsening

**A – English version**

Suppose you are suffering from a chronic, terminal respiratory disease. At home, you need oxygen therapy all the time and your movements are limited. You know that in case of sudden deterioration (for example because of a lung infection), you may have to be put on artificial respiration (a tube connected to a machine that breathes for you, while you are asleep and unconscious), without you being able to give your opinion. Regarding the decision to use this artificial respiration:

1. In your opinion, who should make this advance decision (at a time when the sudden aggravation has not yet occurred)? (a single answer)

🞎 I would prefer to be left to make my own decision

🞎 I would rather the decision be left to me, after having taken my doctor's advice into consideration

🞎 I would rather decide together with my doctor

🞎 I would prefer to let my doctor decide, once my opinion has been taken into consideration

🞎 I would prefer to let my doctor decide alone

1. Is it important for you that your doctor should discuss this decision with you in advance, in anticipation of a sudden deterioration?

🞎 Yes, absolutely

🞎 Mostly yes

🞎 Neutral

🞎 Mostly no

🞎 No, not at all

1. Do you think it is possible to express an opinion regarding this decision at a time when the situation has not yet arisen?

🞎 Yes, absolutely

🞎 Mostly yes

🞎 Neutral

🞎 Mostly no

🞎 No, not at all

**B – French version**

Supposez que vous souffrez d’une maladie respiratoire chronique et irréversible. A la maison, vous avez besoin d’oxygène en permanence et vos déplacements sont limités. Vous savez qu’en cas d’aggravation brutale (par exemple à cause d’une infection pulmonaire), la question peut se poser de vous mettre sous assistance ventilatoire artificielle (un tube relié à une machine qui respire pour vous, alors que vous êtes endormi et inconscient), sans que vous soyez en état de donner votre avis. Concernant la décision de recourir à cette assistance :

1. Selon vous, qui devrait prendre cette décision par anticipation (alors que la situation d’aggravation brutale n’a pas eu lieu) ? (une seule réponse)

🞎 Je préfère qu’on me laisse décider seul

🞎 Je préfère qu’on me laisser décider, après avoir pris en considération l’avis du médecin

🞎 Je préfère que mon médecin et moi décidions ensemble

🞎 Je préfère laisser mon médecin décider, après avoir pris mon avis en considération

🞎 Je préfère laisser mon médecin décider seul

1. Est-ce important pour vous que votre médecin aborde à l’avance cette décision avec vous, en prévision de la survenue d’une aggravation brutale ? (une seule réponse)

🞎 Oui, tout à fait

🞎 Plutôt oui

🞎 Sans opinion

🞎 Plutôt non

🞎 Non, pas du tout

1. Vous semble-t-il possible de vous prononcer sur cette décision alors que la situation ne s’est pas présentée?

🞎 Oui, tout à fait

🞎 Plutôt oui

🞎 Sans opinion

🞎 Plutôt non

🞎 Non, pas du tout
