# Supplementary material for: Validity of the French version of the Autonomy Preference Index and its adaptation for patients with advanced cancer
Source: PLoS One. 2020 Jan 14;15(1):e0227802. doi: 10.1371/journal.pone.0227802 (PMC6959662; doi:10.1371/journal.pone.0227802)
Supplement: S1 Table — (DOCX) [file pone.0227802.s003.docx]

**Supporting information S1**

**S1-Table:** Frequency (%) of the answers to each item of the Autonomy Preference Index in both samples

**1 – Decision making preference scale**

| **Item** |  | **Strongly disagree** | **Disagree** | **Neutral** | **Agree** | **Strongly agree** | **Missing** |
| --- | --- | --- | --- | --- | --- | --- | --- |
| 1* - The important medical decisions should be made by your doctor, not by you. | GP | 26 (7) | 62 (16) | 105 (27) | 131 (33) | 67 (17) | 0 (0) |
|  | ONCO | 22 (12) | 39 (21) | 39 (21) | 43 (23) | 43 (23) | 1 (0) |
| 2* - You should go along with your doctor’s advice even if you disagree with it. | GP | 5 (1) | 46 ( 12) | 75 (19) | 205 (53) | 60 (15) | 0 (0) |
|  | ONCO | 7 (4) | 20 (11) | 40 (21) | 77 (41) | 41 (22) | 2 (1) |
| 3* - When hospitalized, you should not be making decisions about your own care. | GP | 60 (15) | 125 (32) | 76 (19) | 94 (24) | 36 (9) | 0 (0) |
|  | ONCO | 25 (13) | 66 (35) | 27 (14) | 41 (22) | 25 (13) | 3 (2) |
| 4 - You should feel free to make decisions about everyday medical problems. | GP | 5 (1) | 25 (6) | 44 (11) | 213 (54) | 104 (27) | 0 (0) |
|  | ONCO | 2 (1) | 11 (6) | 32 (17) | 98 (52) | 44 (24) | 0 (0) |
| 5* - If you were sick, as your illness became worse you would want your doctor to take greater control. | GP | 7 (2) | 24 (6) | 44 (11) | 223 (57) | 93 (24) | 0 (0) |
|  | ONCO | 2 (1) | 12 (6) | 27 (14) | 103 (55) | 41 (22) | 2 (1) |
| 6 - You should decide how frequently you need a check-up. | GP | 14 (4) | 99 (25) | 93 (24) | 142 (36) | 43 (11) | 0 (0) |
|  | ONCO | 26 (14) | 66 (35) | 49 (26) | 36 (19) | 10 (5) | 0 (0) |

**2 – Clinical vignettes**

A – Upper respiratory tract illness

| **Item** |  | **You alone** | **Mostly you** | **Doctor and you equally** | **Mostly the doctor** | **The doctor alone** | **Missing** |
| --- | --- | --- | --- | --- | --- | --- | --- |
| 7* - Whether you should be seen by a doctor | GP | 111 (28) | 143 (37) | 93 (24) | 32 (8) | 12 (3) | 0 (0) |
|  | ONCO | 32 (17) | 70 (37) | 58 (31) | 17 (9) | 8 (4) | 2 (1) |
| 8* - Whether a chest x-ray should be taken | GP | 8 (2) | 7 (2) | 103 (26) | 185 (47) | 88 (23) | 0 (0) |
|  | ONCO | 2 (1) | 6 (3) | 28 (15) | 103 (55) | 47 (25) | 1 (1) |
| 9* - Whether you should try taking cough syrup | GP | 28 (7) | 46 (12) | 130 (33) | 130 (33) | 57 (15) | 0 (0) |
|  | ONCO | 10 (5) | 22 (12) | 47 (25) | 76 (41) | 31 (17) | 1 (1) |

B – High blood pressure

| **Item** |  | **You alone** | **Mostly you** | **Doctor and you equally** | **Mostly the doctor** | **The doctor alone** | **Missing** |
| --- | --- | --- | --- | --- | --- | --- | --- |
| 10* - When the next visit to check your blood pressure should be. | GP | 14 (4) | 21 (5) | 85 (22) | 181 (46) | 90 (23) | 0 (0) |
|  | ONCO | 2 (1) | 2 (1) | 28 (15) | 93 (50) | 61 (33) | 1 (1) |
| 11* - Whether you should take some time off from your work to relax | GP | 6 (2) | 12 (3) | 147 (38) | 138 (35) | 88 (23) | 0 (0) |
|  | ONCO | 2 (1) | 6 (3) | 55 (29) | 67 (36) | 52 (28) | 5 (3) |
| 12* - Whether you should be treated with medication or diet. | GP | 4 (1) | 19 (5) | 128 (33) | 162 (41) | 78 (20) | 0 (0) |
|  | ONCO | 3 (2) | 2 (1) | 46 (25) | 79 (42) | 56 (30) | 1 (1) |

C – Myocardial infarction

| **Item** |  | **You alone** | **Mostly you** | **Doctor and you equally** | **Mostly the doctor** | **The doctor alone** | **Missing** |
| --- | --- | --- | --- | --- | --- | --- | --- |
| 13* - How often the nurses should wake you up to check your temperature and blood pressure. | GP | 5 (1) | 8 (2) | 32 (8) | 158 (40) | 188 (48) | 0 (0) |
|  | ONCO | 0 (0) | 1 (1) | 13 (7) | 71 (38) | 100 (53) | 2 (1) |
| 14* - Whether you may have visitors aside from your immediate family. | GP | 48 (12) | 50 (13) | 138 (35) | 106 (27) | 49 (13) | 0 (0) |
|  | ONCO | 20 (11) | 25 (13) | 46 (25) | 56 (30) | 38 (20) | 2 (1) |
| 15* - Whether a cardiologist should be consulted. | GP | 16 (4) | 46 (12) | 170 (44) | 94 (24) | 65 (17) | 0 (0) |
|  | ONCO | 8 (4) | 22 (12) | 81 (43) | 44 (24) | 28 (15) | 4 (2) |

**3 – Information-seeking preference scale**

| **Item** |  | **Strongly disagree** | **Disagree** | **Neutral** | **Agree** | **Strongly agree** | **Missing** |
| --- | --- | --- | --- | --- | --- | --- | --- |
| 16 - As you become sicker you should be told more and more about your illness | GP | 2 (1) | 4 (1) | 12 (3) | 127 (32) | 246 (63) | 0 (0) |
|  | ONCO | 1 (1) | 3 (2) | 7 (4) | 60 (32) | 115 (62) | 1 (1) |
| 17 - You should understand completely what is happening inside your body as a result of your illness | GP | 0 (0) | 2 (1) | 8 (2) | 106 (27) | 275 (70) | 0 (0) |
|  | ONCO | 3 (2) | 1 (1) | 10 (5) | 49 (26) | 123 (66) | 1 (1) |
| 18 - Even if the news is bad, you should be well informed | GP | 1 (0) | 2 (1) | 12 (3) | 98 (25) | 278 (71) | 0 (0) |
|  | ONCO | 3 (2) | 2 (1) | 10 (5) | 54 (29) | 117 (63) | 1 (1) |
| 19 - Your doctor should explain the purpose of your laboratory tests | GP | 0 (0) | 0 (0) | 7 (2) | 127 (32) | 257 (66) | 0 (0) |
|  | ONCO | 3 (2) | 0 (0) | 5 (3) | 80 (43) | 97 (52) | 2 (1) |
| 20* - You should be given information only when you ask for it | GP | 95 (24) | 159 (41) | 43 (11) | 60 (15) | 34 (9) | 0 (0) |
|  | ONCO | 50 (27) | 77 (41) | 22 (12) | 29 (16) | 8 (4) | 1 (1) |
| 21 - It is important for you to know all the side effects of your medication | GP | 3 (1) | 4 (1) | 11 (3) | 125 (32) | 248 (63) | 0 (0) |
|  | ONCO | 1 (1) | 0 (0) | 7 (4) | 46 (25) | 132 (71) | 1 (1) |
| 22 - Information about your illness is as important to you as treatment | GP | 2 (1) | 10 (3) | 15 (4) | 141 (36) | 223 (57) | 0 (0) |
|  | ONCO | 5 (3) | 12 (6) | 13 (7) | 56 (30) | 100 (53) | 1 (1) |
| 23 - When there is more than one method to treat a problem, you should be told about each one | GP | 0 (0) | 1 (0) | 10 (3) | 118 (30) | 262 (67) | 0 (0) |
|  | ONCO | 1 (1) | 0 (0) | 3 (2) | 4 (59) | 123 (66) | 1 (1) |

GP: General Practice, ONCO: oncology service, * reversed item to compute the score
