# Supplementary material for: Validity of the French version of the Autonomy Preference Index and its adaptation for patients with advanced cancer
Source: PLoS One. 2020 Jan 14;15(1):e0227802. doi: 10.1371/journal.pone.0227802 (PMC6959662; doi:10.1371/journal.pone.0227802)
Supplement: S2 Table — (DOCX) [file pone.0227802.s004.docx]

**Supporting Information S2**

**S2-Table:** Measurement invariance assessment for the three-factor model of the Autonomy Preference Index

| **Measurement Invariance model**  **(constraints)** | **Chi²**  **(DF)** | **RMSEA**  **(90% CI)** | **CFI** | **TLI** | **ΔRMSEA** | **ΔCFI** |
| --- | --- | --- | --- | --- | --- | --- |
| **Measurement invariance across GP** (N=391) **and ONCO** (N=187) **samples** | | | | | | |
| Configural model | 879.4  (454) | 0.057  (0.051-0.063) | 0.939 | 0.932 | - | - |
| Metric model | 895.1  (474) | 0.055  (0.050-0.061) | 0.940 | 0.936 | -0.002 | 0.001 |
| Scalar model | 982.7  (518) | 0.056  (0.050-0.061) | .934 | 0.935 | 0.001 | -0.006 |
| **Measurement invariance across sex** (Male N=218, Female N=360) | | | | | | |
| Configural model | 910.9  (454) | 0.059  (0.053-0.065) | 0.935 | 0.927 | - | - |
| Metric model | 938.7  (474) | 0.058  (0.053-0.064) | 0.933 | 0.929 | -0.001 | -0.002 |
| Scalar model | 986.1  (518) | 0.056  (0.051-0.061) | 0.933 | 0.935 | -0.002 | 0.000 |
| **Measurement invariance across age** (≤40 y N=147, 41 to 55 y N=146, 56 to 70 y N=182, >70 y N=103) | | | | | | |
| Configural model | 1529.4  (908) | 0.069  (0.063-0.075) | 0.913 | 0.904 | - | - |
| Metric model | 1560.4  (968) | 0.065  (0.059-0.071) | 0.917 | 0.914 | -0.004 | 0.004 |
| Scalar model | 1691.1  (1100) | 0.061  (0.055-0.067) | 0.918 | 0.924 | -0.004 | 0.001 |
| **Measurement invariance across education level** (middle sch. N=106, high sch. N=180, higher N=185) | | | | | | |
| Configural model | 1108.9  (681) | 0.057  (0.051-0.064) | 0.938 | 0.931 | - | - |
| Metric model | 1157.4  (721) | 0.056  (0.050-0.062) | 0.937 | 0.933 | -0.001 | -0.001 |
| Scalar model | 1287.0  (809) | 0.056  (0.050-0.061) | 0.930 | 0.935 | 0.000 | -0.007 |
| **Measurement invariance across languages*** (French N= 578, English N=120) | | | | | | |
| Configural model | 504.2  (152) | 0.081  (0.074-0.089) | 0.955 | 0.946 | - | - |
| Metric model | 506.5  (164) | 0.077  (0.070-0.085) | 0.956 | 0.951 | -0.004 | 0.001 |
| Factorial model | 591.4  (188) | 0.078  (0.071-0.086) | 0.948 | 0.950 | 0.001 | -0.001 |

Configural model: same factor structure hypothesized in the different groups with freely estimated factor loadings and item thresholds in every groups (unconstrained). Metric model: factor loadings constrained to be equal across groups, no constraint on item thresholds. Scalar model: factor loadings and item thresholds constrained to be equal across groups. DF: degrees of freedom, RMSEA: Root Mean Square Error Approximation, 90%CI: 90% confidence interval, CFI: Comparative Fit Index, TLI: Tucker-Lewis Index. ΔCFI and ΔRMSEA refer to the difference between the unconstrained model and the preceding (constrained) one. Each level of measurement invariance was considered to be present if the fit indices difference, ΔCFI and ΔRMSEA, between nested models was –0.01 and 0.015 or below respectively. * two-factor model without clinical vignettes
