# Supplementary material for: Validity of the French version of the Autonomy Preference Index and its adaptation for patients with advanced cancer
Source: PLoS One. 2020 Jan 14;15(1):e0227802. doi: 10.1371/journal.pone.0227802 (PMC6959662; doi:10.1371/journal.pone.0227802)
Supplement: Attachment — Submitted filename: Review_PlosOne.docx [file pone.0227802.s007.docx]

The article Assessment of information preferences and desire to participate in decision-making: validity of the French version of the Autonomy Preference Index and its adaptation for patients with advanced cancer targets the validation of the Autonomy Preference Index (API). I highly appreciate such validation studies that apply confirmatory factor analysis and invariance testing. Although I see problems several major problems in the structure of the manuscript and the conclusion and handling of the results. In the next sections I further describe problems of the paper in detail.

**Title:** In my opinion the title is too long and should be shortened.

**Abstract:** I think the background should also include a quick description why such a validity study and enlargement is necessary. For me it is not clear what is meant by “a consensual French version”. In the results it should be indicated if the three-factors were correlated. As the last sentence in the results sections indicates that one new items was not correlated at all with the API, I believe the conclusion that additional items are of interest for the API is wrong, as this items clearly does not belong to the scale.

Regarding the **open data statement:** I don´t believe this is correct. Why are you not providing the data anonymized? Anonymized data can be shared and I would also like to so the scripts for analysis shared (e.g., in the OSF).

**Introduction:**

73ff you describe three questionnaires. You clearly choose API over the others for your validation study. I would appreciate a description why you choose this over the others.

In my opinion the introduction is too short and misses information about results from previous application of the API (e.g., The results of study [26] should be provided and discussed). This would help the reader to understand why you have chosen the API, why you aim a validity study and why you have developed additional items. Besides, I would like to read more about possible invariance in the introduction. After reading the introduction it is not clear, why you would (not) expect measurement invariance for gender, setting and age. A through explanation for that in the introduction (supported by previous results) is needed to understand the necessity and the aim of the study. I think the introduction needs a clear and literature based expansion on all the above named issues in order to be publishable. Also I highly recommend to at least provide research aims that clearly indicates what was assessed and expected (validation, reliability, invariance, etc.).

**Methods:**

L 102: ) is missing after HBP

As the term vignette can be understand differently it would be helpful if you present an example of the items you invented additionally.

I appreciate the process and description of translation.

2.3 Study samples describe the sample recruitment. What I miss in this section is a clrea description of the sample. How many participants were recruited in total? How is the age, gender, settings and educational level in this sample? Did the participants receive any incentive? How is further data distributed (health status etc.). This is all missing in the methods section and should be in detail included here.

L 159: what is qualitative data? Please elaborate further. I do not understand what of your data can/would be qualitative data (maybe due to confusing/inadequate description of the measurements: the measurement description is distributed all over the methods part and there is no general overview).

L 167: please state how many missing data you have and why you have missing data.

L 172ff: please indicate if the factors were correlated or not.

L 181: a short description of invariance would help reader that are not familiar with invariance to understand what the three invariance types are and how they are tested. I recommend a detail description here.

L 184: please rather indicate Mc Donalds omega (1999) as a reliability coefficient as it is a better estimate than Cronbachs alpha and can be directly estimated in the confirmatory models.

L 190ff: I do not understand how this (age, educational level, etc.) should be indicators for convergent and discriminant validity. Based on its definition a test for convergent validity requires a high correlation with a test that measures arguably the same construct while discriminant validity is indicated by a low correlation with a test that measures a different construct. Please re-consider if these are the correct indicators for testing convergent and discriminant validity – I don´t think so, but I am also no clinical expert – and if so please state why these indicators are correct and how they were testes (normally based on correlations, your test is not clearly understandable for me at this point). I also recommend quickly explaining convergent and discriminant validity.

**Results:**

Table 1 would rather be a good table for the methods section in order to describe the sample. I also don´t understand what the p-value in table 1 implicates. This clearly needs further explanation. I also do not really understand what the N is for API score, and the global judgments. Does the N displays the amount of people that have answered at all? That have answered in a specific way?

L 206: I would also highly appreciate to read more in the introduction if the different types of cancer might lead to differences in answering the questionnaire.

L 216: see my comment above. Why are there any missings – please describe?

In my opinion table 2 would be easier to understand as a graph (e.g. bar chart etc.) for each of the vignettes.

Figure 1. First, the picture quality is quite bad. Please indicate if all loadings were significantly. Was the correlation between decision making and information seeking (.10) really significant? As the introduction is quite vague and missing a lot of information for me it is not clear if the low/n.s. correlation between information seeking and the other scales is expected and how this was modeled before. Anyways from a psychometrical point of view this indicates that the three scales are not measuring the same thing or at least information seeking is something different than the other two scales. Hence I would highly recommend a careful reconstruction of the questionnaire. I recommend deleting items with very low loadings (e.g., below .30) and reconsider the third scale and its meaning for the API (as statistically it has nothing to do with the other scales). If that is expected or clinical meaningful in some way than it has to be made clear in the introduction.

L 231ff: I appreciate that it was tested for measurement invariance. Although due to the somehow confusing methods section it is not clear for me a) how big the single groups are and b) what cut-off were made (e.g., What cutoff was applied for age? And how was that cutoff selected). That has to be clearly stated.

L 244: as said above I highly recommend the use of McDonalds omega instead of alpha.

L 251: what is concurrent validity? This term is new here and was not introduced before. Why are you reporting a priori hypothesis down here, if you don´t report them in the end of the introduction? Please be consistent here.

Table 3. Due to the lacks in the introduction and methods I have problems of following what is what here. Where do I find the three scales of figure 1 in this table? Why is the clinical vignette now divided in three other scales even though it was one scale in figure 1. I highly recommend a better overview (as already stated) and consistent labeling. Besides, this table should be referenced before the invariance testing (maybe even as descriptive statistics in the methods) as I guess these are the groups you refer to in the invariance testing?

L 263ff: I don´t see any relationships in the table but rather quite un-informative descriptive statistics again. Please report correlations – I also recommend reporting them as heat maps to better visualize. Besides, as said above, please use consistent labeling.

L264ff: where is this relation you are talking about? How big are they? Please state *r* and *p*.

**Discussion:**

L277: For this conclusion I need to see a re-considered scale or a very good theoretical explanation why information seeking is not correlated with the other factors.

L279ff: you do not show that? How did you proved incremental validity of your vignette? Please state that differently or provide prove of any incremental validity over the existing scale.

L 285ff: I do not see the benefit of a vignette that is not correlated with nothing. This is no prove for incremental validity! It is not clear what this vignette is measuring in a psychometric way and I would highly recommend to re-consider its use at all.

L287ff: why did you thought that? Any empirical proof (please cite it here) or just a feeling?

L296ff: why? Please elaborate on how this vignette can do that. From a psychometrical point of view I rather see it questionable (due to its correlational results) and doubt its meaning.

L310ff: I appreciate that you mention loading problems and the low correlations between the subscales. But I think this really implies further analysis. The reason not to dig too deep into modelling issue because of “adapting the classic version” seems quite weak. Arguably the classic version comes along with problems (or at least some items have major problems). So why not improve the scale in order to measure the construct even better, more valide and more reliable?
