# Supplementary material for: Validity of the French version of the Autonomy Preference Index and its adaptation for patients with advanced cancer
Source: PLoS One. 2020 Jan 14;15(1):e0227802. doi: 10.1371/journal.pone.0227802 (PMC6959662; doi:10.1371/journal.pone.0227802)
Supplement: Attachment — Submitted filename: APIvalid_ResponseToReviewers.docx [file pone.0227802.s008.docx]

**POINT-BY-POINT REPLY**

Please include the following items when submitting your revised manuscript:

- A rebuttal letter that responds to each point raised by the academic editor and reviewer(s). This letter should be uploaded as separate file and labeled 'Response to Reviewers'.

⭢ **The present document is the rebuttal letter labeled “APIvalid_ResponseTo Reviewers.docx”**

- A marked-up copy of your manuscript that highlights changes made to the original version. This file should be uploaded as separate file and labeled 'Revised Manuscript with Track Changes'.

⭢ **The marked-up copy of the manuscript has been labeled “APIvalid_RevisedManuscriptWithTrackChanges.docx”.**

- An unmarked version of your revised paper without tracked changes. This file should be uploaded as separate file and labeled 'Manuscript'.

⭢ **The unmarked version of the manuscript has been labeled “APIvalid_RevisedManuscript.docx”.**

Journal Requirements:

When submitting your revision, we need you to address these additional requirements.

1. Please ensure that your manuscript meets PLOS ONE's style requirements, including those for file naming. The PLOS ONE style templates can be found at <http://www.journals.plos.org/plosone/s/file?id=wjVg/PLOSOne_formatting_sample_main_body.pdf> and <http://www.journals.plos.org/plosone/s/file?id=ba62/PLOSOne_formatting_sample_title_authors_affiliations.pdf>

**→ Corrections have been done to meet PLOS ONE’s style requirements**

2. We note that you have indicated that data from this study are available upon request. PLOS only allows data to be available upon request if there are legal or ethical restrictions on sharing data publicly. For information on unacceptable data access restrictions, please see <http://journals.plos.org/plosone/s/data-availability#loc-unacceptable-data-access-restrictions>.

In your revised cover letter, please address the following prompts:

a) If there are ethical or legal restrictions on sharing a de-identified data set, please explain them in detail (e.g., data contain potentially identifying or sensitive patient information) and who has imposed them (e.g., an ethics committee). Please also provide contact information for a data access committee, ethics committee, or other institutional body to which data requests may be sent.

b) If there are no restrictions, please upload the minimal anonymized data set necessary to replicate your study findings as either Supporting Information files or to a stable, public repository and provide us with the relevant URLs, DOIs, or accession numbers. Please see <http://www.bmj.com/content/340/bmj.c181.long> for guidelines on how to de-identify and prepare clinical data for publication. For a list of acceptable repositories, please see <http://journals.plos.org/plosone/s/data-availability#loc-recommended-repositories>.

We will update your Data Availability statement on your behalf to reflect the information you provide.

**⭢ The minimal anonymized data set is provided in the file “APIvalid_Data.csv” and variables’ name and labels in the file “APIvalid_Variables.xls”**

Additional Editor Comments (if provided):


Reviewers' comments:

**Reviewer #1**: Very interesting piece of research and rigorously presented. Below some comments:

Line 131: may you provide more information on medical data of the ONCO sample: years since diagnosis, type of treatments received (chemo, radio, surgery...); otherwise did you collect any measure of QoL, physical functioning and mood status?

⭢ **As it was also asked by the reviewer 2, we moved the description of the samples from the “Results” section to the “Methods” section (line 221-231). You will see in this description that “***In the ONCO sample, cancer had been diagnosed for a median time of 20 (8 – 41) months and the primary tumour sites were lung, colon and/or rectum, pancreas and ovary for 47(25%), 27(14%), 23(12%) and 22(12%) of the patients respectively”***. We did not collect the type of treatments received neither measure of QoL. For the physical functioning, we only collected if the ECOG performance status was below 3 as it was an inclusion criterion, so we do not have any other details. Concerning mood status, we used the Hospital Anxiety and Depression Scale in the ONCO sample but not in the GP sample as we did not have enough time to collect it in the GP waiting rooms.**

If not, I miss some discussion about how differences on these aspects may impact on preferences for DM, IS and preparedness to anticipate disease worsening... May this be a limitation regarding the generability of your findings?

⭢ **A specific paper on the relation between DM, IS, preparedness to anticipate disease worsening and the characteristics of the patients in the ONCO sample is being written. Indeed, we think that it would be confusing to add information about these relations in the present paper which aimed at studying the psychometric properties of the API. You are right that characteristics of the patients may have an effect on the psychometric properties of the API, specifically if there is a lack of measurement invariance according to one or several of these characteristics. We have no arguments supporting the hypothesis that it could happen with the diagnosis, type of treatments received, QoL or physical functioning (neither from literature on the topic, nor from our clinical experience). However, for mood status, we agree with you, we can imagine that someone with a high level of depression may interpret some items of the API quite differently than somebody without depression. This cannot be studied in our sample as there was not enough patients with a score higher than 10 at the depression scale of the HADS (N=21) and as HADS was not collected in the GP sample. We added two sentences on this point in the “Discussion” section (line 455-459): *“****Finally, it would have been interesting to assess measurement invariance according to other characteristics, like for example anxiety and depression which may influence the interpretation of some items of the API. However, due to time constraints we did not collect information on depression and anxiety level in the GP sample.”*

Line 141: may you enhance description of how and by who patients from the ONCO sample were approached for data collection (what specialist consultation, known or unknown researcher to the patient...)?

⭢ **We added precisions on the recruitment of the patients in the ONCO sample in the “Methods” section (line 192-199) “***2) in the oncology outpatient clinic of Cochin Hospital in Paris (ONCO sample). Cochin hospital is a tertiary care hospital treating around 4500 new cancer patients each year, with an oncology ward and three other medical specialty wards (gastroenterology, pneumology, dermatology) that have an oncologic activity of care and use the oncology outpatient clinic for ambulatory anticancer treatment and follow-up. Explanations on the study were provided to all consecutive French-speaking patients aged 18 years or older, without cognitive or psychopathologic disorders, by an independent researcher, unknown to the patients in the two settings.”*

Line 245: 8 'DM' items is maybe a typo, I think you mean IS

⭢ **Yes, thank you, we corrected it**

Line 291: I dont quite understand what you mean by 'disrupt coping mechanisms...', in turn I find this idea interestingly enough to develop it a bit further...

⭢ **We added precisions on this point in the “Discussion” section (line 392-395)** *“ Indeed, results from the Coping with Cancer study suggested that patients with such psychosocial factors as emotional numbness may have their fears rather exacerbated by end-of-life discussions, resulting in unreasonable demands of care and life-maintaining treatments [49].”*

Line 292: 'educational initiatives' mean from the patient perspective, like psychoeducation, or from the healthcarer perspective, as in medical school programs?

⭢ **We added precisions on this point in the “Discussion” section (line 395-401)***: “educational initiatives (…) are therefore needed and are currently being developed, in protocols interestingly involving both healthcarers and patients/caregivers perspectives [50–52]”*

**Reviewer #2**: The article Assessment of information preferences and desire to participate in decision-making: validity of the French version of the Autonomy Preference Index and its adaptation for patients with advanced cancer targets the validation of the Autonomy Preference Index (API). I highly appreciate such validation studies that apply confirmatory factor analysis and invariance testing. Although I see problems several major problems in the structure of the manuscript and the conclusion and handling of the results. In the next sections I further describe problems of the paper in detail.

Title: In my opinion the title is too long and should be shortened.

⭢ **Alright, we shortened it (line 1)** *“ Validity of the French version of the* *Autonomy Preference Index and its adaptation for patients with advanced cancer*.”

Abstract: I think the background should also include a quick description why such a validity study and enlargement is necessary.

⭢ **We added precisions on this point in the abstract (line 21-24): “***In oncology, anticipation of disease worsening remains difficult, especially when patient’s preferences towards prognosis medical information are unclear. Valid tools to explore patients’ preferences could help targeting end-of-life discussions, which have been shown to decrease aggressiveness of end-of-life care.”*

For me it is not clear what is meant by “a consensual French version”.

⭢ **We modified it as follows (line 29): “***The psychometric properties of the API translated into French were assessed among patients consecutively recruited from January to March 2017 in the waiting rooms of 19 general practitioners (N=391) and in an oncology (N=187) clinic in Paris.”*

In the results it should be indicated if the three-factors were correlated.

⭢ **We added this information (line 33-34): “***A three-factor confirmatory factor model (two correlated factors related to decision-making and an uncorrelated factor related to information-seeking preferences) showed an acceptable fit on the whole sample and no measurement invariance issue was found across settings, age, sex and educational level*.*”*

As the last sentence in the results sections indicates that one new items was not correlated at all with the API, I believe the conclusion that additional items are of interest for the API is wrong, as this items clearly does not belong to the scale.

⭢ **We do not say that these items belong to the API scale (and we did not any analyses to prove that), we only say that these items add information which is not contained in the API as one new item is not related to the API subscale scores (so to the constructs assessed by the API). So, in the context of incurable illness, these items are of interest to ensure that patient values guide all end-of-life clinical decisions as it is not assessed by the API and in any other scale to our knowledge.**

Regarding the open data statement: I don´t believe this is correct. Why are you not providing the data anonymized? Anonymized data can be shared and I would also like to so the scripts for analysis shared (e.g., in the OSF).

**⭢ The minimal anonymized data set is provided in the file “APIvalid_Data.csv” and variables’ name and labels in the file “APIvalid_Variables.xls**

Introduction:
73ff you describe three questionnaires. You clearly choose API over the others for your validation study. I would appreciate a description why you choose this over the others.

⭢ **We added precisions on this point in the “Introduction” section (line 85 to 105)** *“The API has various advantages to be used among patients with incurable cancer over the two other measurement tools identified. First, it does not focus on self-medication contrary to the Krantz Health Opinion Survey. Second, its psychometric properties have already been studied in English and German in various populations (primary care settings, patients with asthma, mental illness, chronic pain) [20–25]. Third, its original structure allows for adaptation depending on the context as it has already been done for psychiatric patients for example* *[21,24,25]. Among the 23 items of its original version, 8 items assess information-seeking (IS score) preferences and the remaining 15 items assess preferences for participation in decision-making (DM) including 6 general items used to compute the DM score and 9 items related to three clinical vignettes representing different levels of severity : the upper respiratory tract illness (URI score) is used to represent a mild condition; hypertension (HBP score) for a moderately severe condition; and myocardial infarction (MI score) for a severe life-threatening condition. In some previous studies, only the 14 items related to the IS and DM scores were used [23,25,26] while in others, vignettes were adapted to the context [20,21,24]”*

In my opinion the introduction is too short and misses information about results from previous application of the API (e.g., The results of study [26] should be provided and discussed).

**In the study [26], which aimed to evaluate an intervention facilitating end-of-life discussions in a randomized clinical trial, the API was used “to assess the baseline similarity between groups as well as between participants who remained in the study versus those who were lost to questionnaire follow-up”. Unfortunately, of the paper and authors only mention in the Results section that: “The groups were well matched at baseline, with no differences on demographic variables (Table 1). Participants who withdrew or died were older (P .03), preferred a more passive role in decision making (P .04)” and no detailed result of the API is reported in the Table 1. The results of the trial itself do not bring any relevant information in introduction of our objective.**

This would help the reader to understand why you have chosen the API, why you aim a validity study and why you have developed additional items.

⭢ **We clarified this point in the “Introduction” section (line 105-108)** *“ So in our study, we aimed to translate and to evaluate the psychometric properties of the API in a population of patients with incurable cancer, and to develop supplementary items, specifically for these patients, to evaluate their preparedness to anticipate disease deterioration, as this was not addressed in the original API.”*

Besides, I would like to read more about possible invariance in the introduction. After reading the introduction it is not clear, why you would (not) expect measurement invariance for gender, setting and age. A through explanation for that in the introduction (supported by previous results) is needed to understand the necessity and the aim of the study. I think the introduction needs a clear and literature based expansion on all the above named issues in order to be publishable. Also I highly recommend to at least provide research aims that clearly indicates what was assessed and expected (validation, reliability, invariance, etc.).

⭢ **We reformulated the last paragraph of the “Introduction” section to be more precise this points (line 109-120)** *“ The working objectives of this study was thus to translate the API, to evaluate its psychometric properties (reliability and construct validity) in a population of primary care patients, as for the original version, and in a population of patients with incurable cancer. We also assessed measurement invariance which is an essential property for questionnaire, as for any measurement tool, to guarantee accurate group comparisons. According to Mokkink et al. and Milsap, “a measuring device should function in the same way across varied conditions, so long as those varied conditions are irrelevant to the attribute being measured” [27,28]. We studied measurement invariance across age, sex and education level as usually performed and recommended, across French and English languages to ensure the comparability of the scores from both language versions, and across both settings (primary care patients and patients with incurable cancer) to check the likeness of the API factor structure in these settings [28–30].”*

Methods:
L 102: ) is missing after HBP

⭢ **Thank you, we fixed it**

As the term vignette can be understand differently it would be helpful if you present an example of the items you invented additionally.

⭢ **Yes, you are right, the entire additional vignette and the 3 associated items are presented in French and in English in the Supporting Information “S2-Questionnaire” and in the Table 1 (ex-table 2). It is indicated in the text in the “methods” section (line 145 to 172)**

I appreciate the process and description of translation.

⭢ **Thank you.**

2.3 Study samples describe the sample recruitment. What I miss in this section is a clrea description of the sample. How many participants were recruited in total? How is the age, gender, settings and educational level in this sample? Did the participants receive any incentive? How is further data distributed (health status etc.). This is all missing in the methods section and should be in detail included here.

⭢ **As it was also asked by the reviewer 1, we moved the description of the samples from the “Results” section to the “Methods” section and added the information you asked (line 221-231).**

L 159: what is qualitative data? Please elaborate further. I do not understand what of your data can/would be qualitative data (maybe due to confusing/inadequate description of the measurements: the measurement description is distributed all over the methods part and there is no general overview).

⭢ **You are right, sorry, it is a mistake, it is “Categorical data”, we fixed it (line 233).**

L 167: please state how many missing data you have and why you have missing data.

⭢ **We indicated the percentage of missing data in the “methods” section (line 291-295): “***Analyses were performed using Stata v.14 software for data management and basic statistics and Mplus v7.4 software for the confirmatory factor analysis (CFA), which implements full information maximum likelihood to handle missing data (lower than 2% whatever the item in the whole sample) [34,35].”*

L 172ff: please indicate if the factors were correlated or not.

⭢ **We indicated the correlation between factors in the 3 and 2-factor models previously observed in the literature in the “methods” section (line 243-244): *“****In the previous studies, the factor corresponding to the 8 IS items was not or poorly correlated (<0.3) to the factor(s) related to DM items [19,24,25].”*

L 181: a short description of invariance would help reader that are not familiar with invariance to understand what the three invariance types are and how they are tested. I recommend a detail description here.

⭢ **You are right, we tried to be concise in the main text as recommended by the editor but a detailed description on the 3 invariance types may be important in the “methods” section for the reader so we reproduced the description which was in the legend of the S2-Table in the “methods” section (line 262-268): “***We consecutively tested these three levels of invariance in fitting three different nested models having increasing constraints. For the sex invariance for example, the same model was hypothesized in both groups and the followed sequence of nested model tests was: 1) configural invariance: unconstrained factor loadings and item thresholds; 2) metric invariance: factor loadings constrained to be equal across sex groups and unconstrained item thresholds; 3) scalar invariance: factor loadings and item thresholds constrained to be equal across sex groups.”*

L 184: please rather indicate Mc Donalds omega (1999) as a reliability coefficient as it is a better estimate than Cronbachs alpha and can be directly estimated in the confirmatory models.

⭢ **Yes of course, we added Mc Donald’s omega estimates in the “methods” section (line 272): *“****Internal consistency was assessed using Cronbach’s alpha and McDonald’s omega coefficients (acceptable if ≥0.7) [43,44]”*, **and in the “results” section (line 337-339):** *“McDonald’s omega coefficients were 0.72 for the 6 DM items, 0.73 for the 9 items related to the clinical vignettes and 0.75 for the 8 IS items.”*

L 190ff: I do not understand how this (age, educational level, etc.) should be indicators for convergent and discriminant validity. Based on its definition a test for convergent validity requires a high correlation with a test that measures arguably the same construct while discriminant validity is indicated by a low correlation with a test that measures a different construct. Please re-consider if these are the correct indicators for testing convergent and discriminant validity – I don´t think so, but I am also no clinical expert – and if so please state why these indicators are correct and how they were testes (normally based on correlations, your test is not clearly understandable for me at this point). I also recommend quickly explaining convergent and discriminant validity.

⭢ **Sorry, you’re right, it’s a wrong formulation, according to COSMIN checklist the right formulation is “Hypothesis testing”, we fixed this mistake in the “methods” section (line 278): *“****Finally, for hypothesis testing, mean API subscale scores were compared, using a one-way analysis of variance or Student t-tests as appropriate between patients according to sex (a priori hypothesis: lower scores among men), age (lower scores among older patients), marital status (higher scores for singles) and educational level (higher scores for higher education levels).”*

Results:
Table 1 would rather be a good table for the methods section in order to describe the sample.

⭢ **The Table 1 has been moved in the “methods” section and renamed Table 2 (line 221)**

I also don´t understand what the p-value in table 1 implicates. This clearly needs further explanation.

⭢ **You’re right, it is not necessary to compare the two samples with statistical tests in this study as it is not one of our objectives. We removed the p-value column in the table 2 (ex-table 1) (line 221).**

I also do not really understand what the N is for API score, and the global judgments. Does the N displays the amount of people that have answered at all? That have answered in a specific way?

⭢ **For the API scores, it is not frequencies that are indicated in the Table 2 (ex-table 1) (line 221) but the mean (Standard Deviation) as it is indicated in the subtitle “***Autonomy Preference Index scores - mean (SD)”.* **Concerning the global judgments, the N displays the amount of people who answered each modality. We added a reference to Table 2 (ex-table 1) in the “methods” section so that it will be clearer that responses to these questions are on Likert-scales and how are formulated the response categories (line 217) *:”*** *The patients completed the French version of the API (and the additional vignette in the ONCO sample) and answered two questions on their global judgment concerning their information preferences (on a 4-point Likert scale) and their desire to participate in decisions (on a 5-point Likert scale) (Table 2).”*

L 206: I would also highly appreciate to read more in the introduction if the different types of cancer might lead to differences in answering the questionnaire.

⭢ **As explained to the previous reviewer, a specific paper on the relation between DM, IS, preparedness to anticipate disease worsening and the characteristics of the patients in the ONCO sample is being written. We will in this paper describe the API scores according to the type of cancer but we think that it would be confusing to add information about these relations in the present paper which aimed at studying the psychometric properties of the API.**

L 216: see my comment above. Why are there any missings – please describe?

⭢ **We are not sure to understand your request. We cannot describe why some answers are missing, it can be because the patient did not want to answer, because he/she forgot to answer, because he/she did not understand a word in the item… We are just able to formulate hypotheses as we did not ask to every patient why he/she did not answer to one of the item.**

In my opinion table 2 would be easier to understand as a graph (e.g. bar chart etc.) for each of the vignettes.

⭢ **The table 1 (ex-table 2) concerns only one vignette: the additional vignette we developed to evaluate preparedness to anticipate disease worsening with the 3 related items as explained and clarified following your suggestions in the “methods” section (line 145-172). We already tried before submission to present the description of the answers to each of the 3 items as a graph but as the labels of the response categories are long, particularly for the first item, the size of three graphs is very difficult to homogenize and the finish is not very nice.**

Figure 1. First, the picture quality is quite bad. Please indicate if all loadings were significantly.

⭢ **We have enhanced the quality of the picture in the file “APIvalid_Figure1.tif” and clarified the legend of the figure 1 which is in the “results” section (line 323-329), as recommended in the “recommendations for authors”*: ” Fig 1****.* ***Path diagrams (with standardized coefficients) for the three factor model with fit indices using a confirmatory factor analysis (robust weighted least squares [WLSMV] estimator)****. Ellipses represent unobserved latent factors, rectangles represent observed variables, single-headed arrows represent the effect of one variable on another (factor loading) and double-headed arrows represent covariance between pairs of variables. Coefficients are all statistically significant with a p-value<0.001, except *p-value=0.039 and coefficient in grey which is not statistically significant. ε: measurement error df: degree of freedom. CFI: Comparative Fit Index. TLI: Tucker Lewis Index. RMSEA: Root Mean Square Error Approximation.”*

Was the correlation between decision making and information seeking (.10) really significant?

⭢ **Yes, we indicated the p-value in the legend of the figure 1 in the “results” section (line 328-329)*: ”****Coefficients are all statistically significant with a p-value<0.001, except *p-value=0.039 and coefficient in grey which is not statistically significant.”*

As the introduction is quite vague and missing a lot of information for me it is not clear if the low/n.s. correlation between information seeking and the other scales is expected and how this was modeled before. Anyways from a psychometrical point of view this indicates that the three scales are not measuring the same thing or at least information seeking is something different than the other two scales. Hence I would highly recommend a careful reconstruction of the questionnaire. I recommend deleting items with very low loadings (e.g., below .30) and reconsider the third scale and its meaning for the API (as statistically it has nothing to do with the other scales). If that is expected or clinical meaningful in some way than it has to be made clear in the introduction.

⭢ **We hope that the precisions we provided in the “methods” section concerning the correlations found in previous studies between the IS factor and the DM factors (line 242-244) make it clearer. We agree with you that if we were developing this scale, we would probably have proceeded in another way. However, our aim was to translate and study the psychometric properties of the API so we cannot modify the original scale in removing items or subscales. We agree with you that the IS scale assesses a completely different construct than DM and that it finally suggests that this 8 items could certainly be used independently to the 15 items related to DM. Fortunately, the scoring system is consistent with this result as the IS score is computed independently from the 15 items related to DM. We highlighted these points in the discussion section (line 413-421) *“*** *In agreement with previous findings, the desire for information factor was poorly correlated with the decision-making factor, [19,22,24] and it was the same items (4, 6 and 20; the reversely coded items) that were found to have low loadings [26].* *No further analyses were performed to assess the fit of adapted models (i.e. without these reversely coded items, as in Bonfils et al [26]), as our aim was to adapt the classic version of API into French to facilitate comparisons between studies that have already used this version. However, our results are consistent with those of previous studies and suggest that it would be interesting to carefully reconstruct this instrument to enhance its psychometric properties.”*

L 231ff: I appreciate that it was tested for measurement invariance. Although due to the somehow confusing methods section it is not clear for me a) how big the single groups are and b) what cut-off were made (e.g., What cutoff was applied for age? And how was that cutoff selected). That has to be clearly stated.

⭢ **Sorry for the “confusing methods section”. We have added a lot of information following your suggestions that have enhance the clarity of this section we think. Thank you. In addition to the description of the categorical variables used to study invariance (line 259-261): “***Measurement invariance was tested consecutively across groups defined by the inclusion setting (GP or ONCO sample), age (categorized according to quartiles), sex, educational level and language version.”* **) and to the size of the groups indicated in the table 2 (ex-table 1, line 221) and also in the table 3 (line 354), we added the size of the groups in the S2-Table”** *Measurement invariance assessment for the three-factor model of the Autonomy Preference Index* ***“***

L 244: as said above I highly recommend the use of McDonalds omega instead of alpha.

⭢ **Yes of course, we added Mc Donald’s omega estimates in the “methods” section (line 272): *“****Internal consistency was assessed using Cronbach’s alpha and McDonald’s omega coefficients (acceptable if ≥0.7) [43,44]”*, **and in the “results” section (line 337-339):** *“McDonald’s omega coefficients were 0.72 for the 6 DM items, 0.73 for the 9 items related to the clinical vignettes and 0.75 for the 8 IS items.”*

L 251: what is concurrent validity? This term is new here and was not introduced before.

⭢ **Sorry, it’s an old word for “convergent”, we changed it into “convergent” in the “methods” section (line 276): “***To assess convergent validity, the association between API subscale scores and the patients’ global judgment on their information preferences and desire to participate in decisions was evaluated using a one-way analysis of variance.”* **and in the “results” section (line 345-346): “***Results concerning convergent validity and hypothesis testing are shown in Table 3. Good convergent validity was observed for every subscale with statistically higher scores in groups defined by a stronger desire for decision-sharing and information.*

Why are you reporting a priori hypothesis down here, if you don´t report them in the end of the introduction? Please be consistent here.

⭢ **The *a priori* hypotheses are listed in the “methods” section as recommended by the COSMIN guidelines (line 278-284): *“****. Finally, for hypothesis testing, mean API subscale scores were compared, using a one-way analysis of variance or Student t-tests as appropriate between patients according to sex (a priori hypothesis: lower scores among men), age (lower scores among older patients), marital status (higher scores for singles) and educational level (higher scores for higher education levels).”* **If the reviewer does not mind, we would prefer to let this reporting in the “methods” section as the “introduction” section is rather long now***.*

Table 3. Due to the lacks in the introduction and methods I have problems of following what is what here. Where do I find the three scales of figure 1 in this table? Why is the clinical vignette now divided in three other scales even though it was one scale in figure 1. I highly recommend a better overview (as already stated) and consistent labeling. Besides, this table should be referenced before the invariance testing (maybe even as descriptive statistics in the methods) as I guess these are the groups you refer to in the invariance testing?

⭢ **Sorry for the “lacks in the introduction and methods”. We have added a lot of information following your suggestions that have enhance the clarity of these sections we hope. Thank you. We actually forgot to indicate in the title of table 3 that it concerns convergent validity and hypothesis testing, so we fixed it (line 354-355): “*Table 3****: Convergent validity and hypothesis testing: mean (SD) scores to the API subscales according to patients’ global judgement and characteristics (N=578)”*. **Also, we modified the table 3 to indicate that the columns correspond to the 5 API subscale scores as explained in the “introduction” section (line 91-103) : “** *Among the 23 items of its original version, 8 items assess information-seeking (IS score) preferences and the remaining 15 items assess preferences for participation in decision-making (DM) including 6 general items used to compute the DM score and 9 items related to three clinical vignettes representing different levels of severity : the upper respiratory tract illness (URI score) is used to represent a mild condition; hypertension (HBP score) for a moderately severe condition; and myocardial infarction (MI score) for a severe life-threatening condition.*” **and in the “methods” section (line 138-144): “***A 5-point Likert scale is used to answer to the 23 items of the API (a score of 5 indicating the strongest preference) (S1-Questionnaire, S1-Table). The computation of the five scores from the API was explained in the original publication as follow: The IS and DM scores are computed as the sum of the 8 and 6 answers respectively linearly adjusted to range from 0 to 100 (strongest desire possible). The URI, HBP and MI scores are computed from the sum of the answers to the three items, linearly adjusted to range from 0 to 10 (strongest desire possible)[19].*”

L 263ff: I don´t see any relationships in the table but rather quite un-informative descriptive statistics again. Please report correlations – I also recommend reporting them as heat maps to better visualize. Besides, as said above, please use consistent labeling.

⭢ **As already written, we thank you for your suggestions that have greatly enhance the clarity of the main text of this manuscript. We think that it is now clearer that the table 3 is reporting results concerning convergent and hypothesis testing as explained in the “methods” section (line 276-284): “***To assess convergent validity, the association between API subscale scores and the patients’ global judgment on their information preferences and desire to participate in decisions was evaluated using a one-way analysis of variance. Finally, for hypothesis testing, mean API subscale scores were compared, using a one-way analysis of variance or Student t-tests as appropriate between patients according to sex (a priori hypothesis: lower scores among men), age (lower scores among older patients), marital status (higher scores for singles) and educational level (higher scores for higher education levels).”***. We added in the legend of the Table 3 the test corresponding to each p-value as we forgot to indicate it in the previous version**

L264ff: where is this relation you are talking about? How big are they? Please state r and p.

⭢ **We added in the text in the “Results” section the corresponding p-values that are indicated in the Table 4 to make it clearer (line 361-369): “***The relationships between answers to the items in the additional clinical vignette and the API subscale scores are shown in Table 4. The desire to participate in the advance decisions was strongly and positively related to DM and the vignette subscale scores (p<0.001), but not to the IS score (p=0.223); preference regarding the anticipation of this decision was related to the IS score (p=0.010) but not to other API scores (p>0.05), and the ability to decide on this point at a time when the situation had not yet arisen was not related to any of the API subscale scores (p>0.05).”* **We also added precision in the title of the Table 4 that the API subscale scores are presented as median (quartile 1 – quartile 3) as we forgot it, sorry (line 371).**

Discussion:
L277: For this conclusion I need to see a re-considered scale or a very good theoretical explanation why information seeking is not correlated with the other factors.

⭢ **We hope that with all the precisions we added in the “introduction”, “methods” and “results” sections following your suggestions it is now clearer that the psychometric properties of the French version of the API are adequate in these settings.**

L279ff: you do not show that? How did you proved incremental validity of your vignette? Please state that differently or provide prove of any incremental validity over the existing scale. I do not see the benefit of a vignette that is not correlated with nothing. This is no prove for incremental validity! It is not clear what this vignette is measuring in a psychometric way and I would highly recommend to re-consider its use at all.

⭢ **We think that with all the precisions we added in the “introduction”, “methods” and “results” sections following your suggestions, it is now clearer that the additional vignette brought information on the preparedness to anticipate disease deterioration that is not addressed by any of the API subscales. This is shown in the 4 last lines of the table 4 as explained in the “results” section (line 365-367): “***and the ability to decide on this point at a time when the situation had not yet arisen was not related to any of the API subscale scores (p>0.05).”* **We deliberately never mentioned “Incremental validity” in this paper as we are not in this context. As explained in the “introduction” section (line 93 to 107), the API assesses different constructs : IS and DM preferences but not preparedness to anticipate disease deterioration which is important to assess, particularly in the context of incurable illness. We did not want to enhance the psychometric properties of the API which are already acceptable as shown in this manuscript and in previous researches referenced in the “introduction” section. Another study could be performed ad hoc to reconstruct the API and enhance its psychometric properties but it was not our aim as stated in the “introduction” section. We added a sentence on that point in the “discussion” section (line 419-421): “***However, our results are consistent with those of previous studies and suggest that it would be interesting to carefully reconstruct this instrument to enhance its psychometric properties.* “

L287ff: why did you thought that? Any empirical proof (please cite it here) or just a feeling?

⭢ **We agree that the use of the verb “think” and the thought itself are inaccurate. We rephrased it (Line 387-388):** *“Interestingly, whereas the practice of end-of-life discussions is far from being common in France and very few patients have written their living wills [5], …*

L296ff: why? Please elaborate on how this vignette can do that. From a psychometrical point of view I rather see it questionable (due to its correlational results) and doubt its meaning.

⭢ **See above, the third item of this vignette adds additional information to the information brought by the original API as the answers to the third item is not correlated to the constructs assessed by the API. We think that all the precisions we added in the “introduction”, “methods” and “results” sections following your suggestions, this point is now clearer.**

L310ff: I appreciate that you mention loading problems and the low correlations between the subscales. But I think this really implies further analysis. The reason not to dig too deep into modelling issue because of “adapting the classic version” seems quite weak. Arguably the classic version comes along with problems (or at least some items have major problems). So why not improve the scale in order to measure the construct even better, more valide and more reliable?

⭢ **See above,** **we added a sentence on that point in the “discussion” section (line 419-421): “***However, our results are consistent with those of previous studies and suggest that it would be interesting to carefully reconstruct this instrument to enhance its psychometric properties.* “
