# Supplementary material for: Validity of the French version of the Autonomy Preference Index and its adaptation for patients with advanced cancer
Source: PLoS One. 2020 Jan 14;15(1):e0227802. doi: 10.1371/journal.pone.0227802 (PMC6959662; doi:10.1371/journal.pone.0227802)
Supplement: Attachment — Submitted filename: PONE-D-19-15483R1.docx [file pone.0227802.s009.docx]

Thank you for re-working your paper. I think the paper is much better now, easier to read and shows interesting, valuable and good results. I thank the authors for working on all my prior suggestions.

I have two minor points that has to be addressed prior to publication as I think the authors made a mistake here:

Added information (line 33-34): “A three-factor confirmatory factor model (two correlated factors related to decision-making and an uncorrelated factor related to information-seeking preferences” This is not what you report as final model in figure 1 (these are three correlated factors). Is this the final model you report? I either suggest re-working figure 1 towards your final model (as this is the only model you display it is misleading) if it is not the final model. If figure 1 is the final model you have to re-write the passage accordingly and report three correlated factors. For clarification: the factor information seeking shows only small/no correlation with the other factors. Nevertheless, the model has to be declared as three-correlated factors as you allow the factors to correlate (maybe this was the mistake here?). This indicates: three-correlated factor model that as a result shows that one factor is not/smally correlated with the others.

Added information in Line 243-244: Again the factors were allowed to be correlated but as a result they only showed small/no significant correlation. Please be consistent here.
