# Supplementary material for: Validity of the French version of the Autonomy Preference Index and its adaptation for patients with advanced cancer
Source: PLoS One. 2020 Jan 14;15(1):e0227802. doi: 10.1371/journal.pone.0227802 (PMC6959662; doi:10.1371/journal.pone.0227802)
Supplement: Attachment — Submitted filename: APIvalid_ResponseToReviewers.docx [file pone.0227802.s010.docx]

**POINT-BY-POINT REPLY**

Please include the following items when submitting your revised manuscript:

- A rebuttal letter that responds to each point raised by the academic editor and reviewer(s). This letter should be uploaded as separate file and labeled 'Response to Reviewers'.

⭢ **The present document is the rebuttal letter labeled “APIvalid_ResponseTo Reviewers.docx”**

- A marked-up copy of your manuscript that highlights changes made to the original version. This file should be uploaded as separate file and labeled 'Revised Manuscript with Track Changes'.

⭢ **The marked-up copy of the manuscript has been labeled “APIvalid_RevisedManuscriptWithTrackChanges.docx”.**

- An unmarked version of your revised paper without tracked changes. This file should be uploaded as separate file and labeled 'Manuscript'.

⭢ **The unmarked version of the manuscript has been labeled “APIvalid_RevisedManuscript.docx”.**

Review Comments to the Author

Reviewer #2: Thank you for re-working your paper. I think the paper is much better now, easier to read and shows interesting, valuable and good results. I thank the authors for working on all my prior suggestions.
I have two minor points that has to be addressed prior to publication as I think the authors made a mistake here:
Added information (line 33-34): “A three-factor confirmatory factor model (two correlated factors related to decision-making and an uncorrelated factor related to information-seeking preferences” This is not what you report as final model in figure 1 (these are three correlated factors). Is this the final model you report? I either suggest re-working figure 1 towards your final model (as this is the only model you display it is misleading) if it is not the final model. If figure 1 is the final model you have to re-write the passage accordingly and report three correlated factors. For clarification: the factor information seeking shows only small/no correlation with the other factors. Nevertheless, the model has to be declared as three-correlated factors as you allow the factors to correlate (maybe this was the mistake here?). This indicates: three-correlated factor model that as a result shows that one factor is not/smally correlated with the others.

⭢ **Yes you are right, we fixed this mistake in the abstract as the model depicted in the figure 1 is the final model (line 31-34) : “***A three correlated factors confirmatory model (two factors related to decision-making and a factor related to information-seeking preferences) showed an acceptable fit on the whole* sample *and no measurement invariance issue was found across settings, age, sex and educational level.*”

Added information in Line 243-244: Again the factors were allowed to be correlated but as a result they only showed small/no significant correlation. Please be consistent here.

 ⭢ **We also modified this sentence to be more consistent (line 357-359) : “***In agreement with previous findings, the desire for information factor was not or poorly correlated with the decision-making factors, [19,21,23] and it was the same items (4, 6 and 20; the reversely coded items) that were found to have low loadings [25].”*

While revising your submission, please upload your figure files to the Preflight Analysis and Conversion Engine (PACE) digital diagnostic tool, <https://pacev2.apexcovantage.com/>. PACE helps ensure that figures meet PLOS requirements. To use PACE, you must first register as a user. Registration is free. Then, login and navigate to the UPLOAD tab, where you will find detailed instructions on how to use the tool. If you encounter any issues or have any questions when using PACE, please email us at [figures@plos.org](mailto:figures@plos.org). Please note that Supporting Information files do not need this step.

  ⭢ **We used PACE to ensure that the Figure 1 (APIvalid_Figure1.tif) meet PLOS requirements.**
